# Recurrent Merkel cell carcinoma of the testis with unknown primary site: a case report

**DOI:** 10.1186/s13256-016-1102-5

**Published:** 2016-11-05

**Authors:** Angela Mweempwa, Alvin Tan, Michael Dray

**Affiliations:** 1 Medical Oncology, Waikato Hospital, Selwyn Street and Pembroke Street, Hamilton, 3204 New Zealand; 2 Histology Department, Waikato Hospital, Selwyn Street and Pembroke Street, Hamilton, 3204 New Zealand

**Keywords:** Testicular, Merkel cell carcinoma, Case report, Chemotherapy, Radiotherapy

## Abstract

**Background:**

Merkel cell carcinoma is a rare and aggressive neuroendocrine tumor that commonly arises in the skin. It is rare for it to occur in the testes. There are only seven cases of testicular Merkel cell carcinoma reported in the literature.

**Case presentation:**

A 66-year-old Maori man presented to our hospital with left testicular swelling. His alpha-fetoprotein and beta-human chorionic gonadotrophin levels were within normal limits. His lactate dehydrogenase concentration was elevated to 267 U/L. Ultrasound imaging confirmed a large testicular mass, and he underwent left orchiectomy. His histological examination revealed a neuroendocrine tumor with an immunostaining pattern suggesting Merkel cell carcinoma. He presented to our hospital again 3 months later with right testicular swelling that was confirmed on ultrasound sonography to be a tumor. He underwent a right orchiectomy, and his histological examination revealed metastatic Merkel cell carcinoma. A primary lesion was not identified, and computed tomographic imaging did not reveal spread to other organs. He received six cycles of adjuvant carboplatin and etoposide chemotherapy and remained disease-free 18 months after completion of chemotherapy.

**Conclusions:**

Given the paucity of studies, standard adjuvant treatment for testicular Merkel cell carcinoma remains uncertain, although platinum-based chemotherapy seems to be an appropriate option.

**Electronic supplementary material:**

The online version of this article (doi:10.1186/s13256-016-1102-5) contains supplementary material, which is available to authorized users.

## Background

Merkel cell carcinoma (MCC) is a rare and aggressive neuroendocrine malignancy that often arises in sun-exposed skin and predominantly affects the elderly. The mean ages at diagnosis are 76 years for women and 74 years for men [1]. Immunosuppression caused by solid organ transplant, human immunodeficiency virus (HIV) infection, and B-cell lymphoproliferative malignancies is associated with an increased risk of MCC [2–4]. Although originally thought to arise from Merkel cells in the skin, it has been suggested that MCC may instead originate from skin stem cells or pro-/pre- and pre-B cells [5, 6]. Merkel cell polyoma virus (MCPyV), a double-stranded deoxyribonucleic acid (DNA) virus, has been linked to the development of MCC [7] and may have a role to play in the transformation of pro-/pre- and pre-B cells into MCC.

MCC more commonly occurs in the skin of the head and neck, followed by the upper limbs and shoulders. It is uncommon for MCC to have an unknown primary site [1]. MCC metastasizing to the testis is rare. To date, there are seven cases of testicular MCC published in the literature [8–13]. Clinical details of all cases are summarized in Additional file 1: Table S1.

## Case presentation

**Fig. 1:**
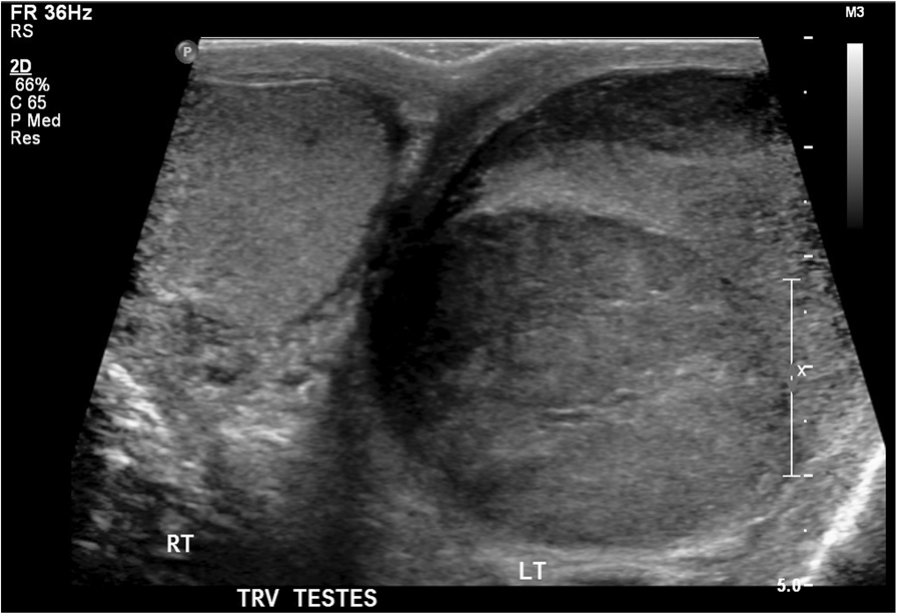
Ultrasound image of the patient’s left testis. The left testis is replaced by a large heterogenous mass

**Fig. 2:**
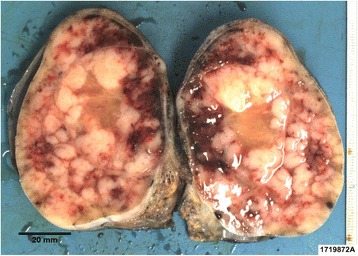
Macroscopic image of the left testis. The left testis consists of well-circumscribed nodular lesions of varying sizes containing solid and gelatinous components. Scale bar = 20 mm

**Fig. 3:**
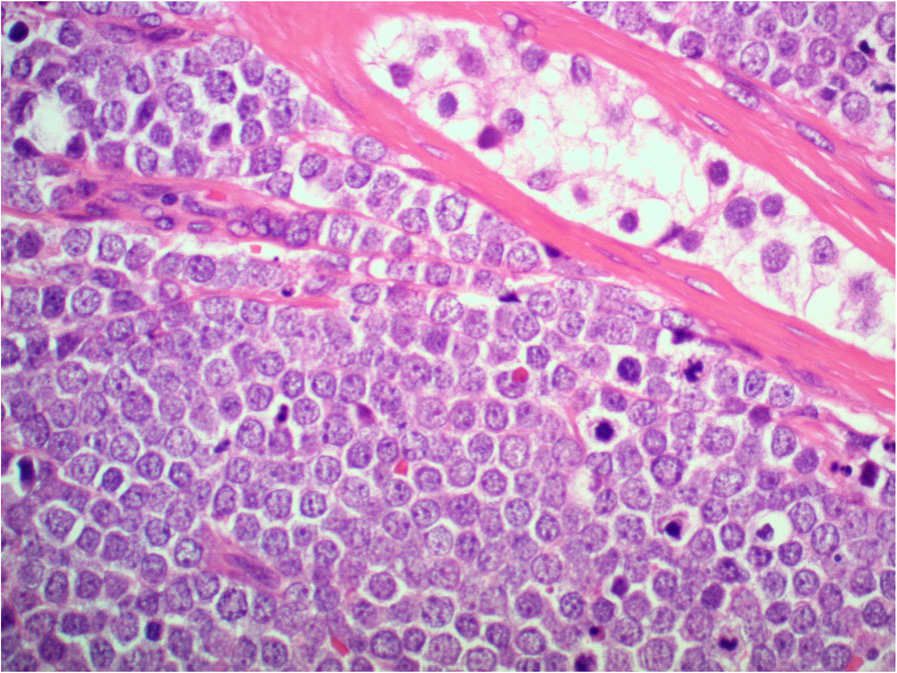
High-power image (hematoxylin and eosin-stain, original magnification ×400) showing small, blue, round cells. Tumor nuclei have a neuroendocrine appearance with speckled chromatin. A residual seminiferous tubule is seen at *top right*

**Fig. 4:**
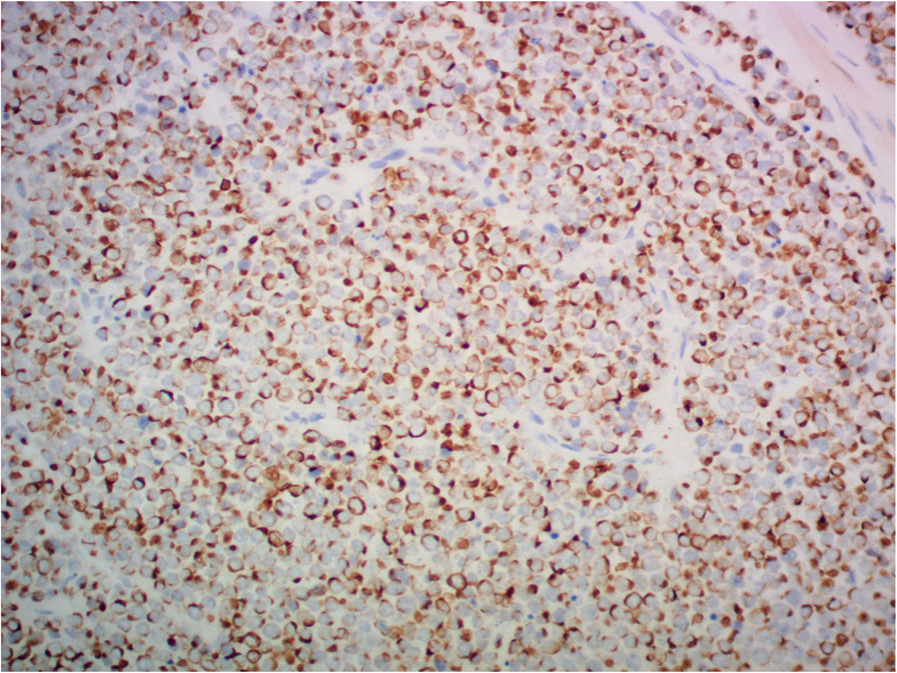
Positive result for cytokeratin 20 with a typical dotlike pattern of staining (original magnification ×200)

**Fig. 5:**
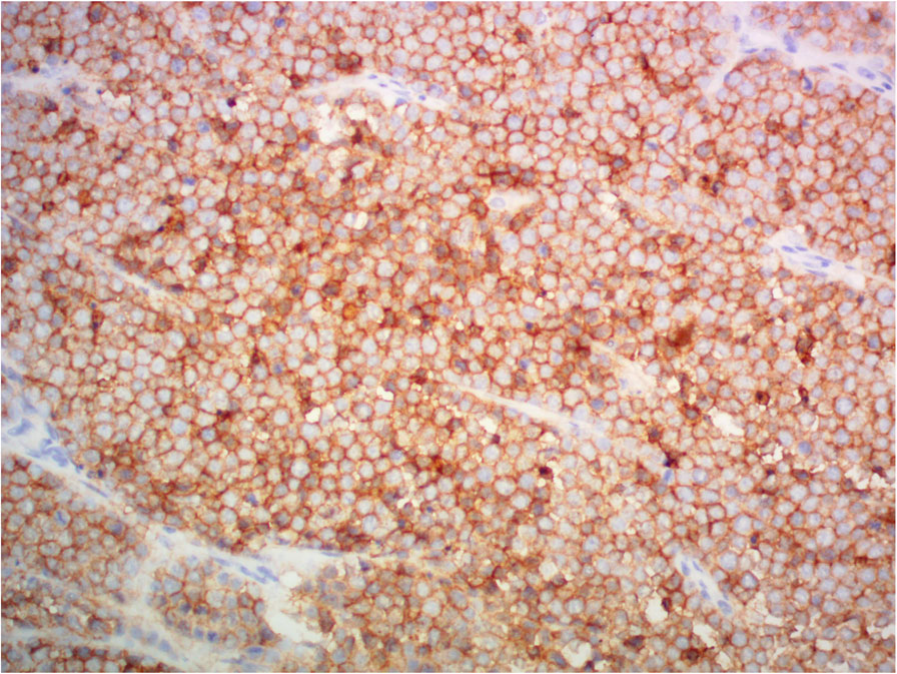
Positive result for CD56, a neuroendocrine marker (original magnification ×200)

A 66-year-old Maori man presented to our hospital with a history of an enlarged left testicle of 10 weeks’ duration. His clinical examination revealed nontender swelling of the left testicle. His alpha-fetoprotein (AFP) and beta-human chorionic gonadotropin (BHCG) levels were within normal limits. His lactate dehydrogenase (LDH) concentration was mildly elevated at 267 U/L (normal range 120–250 U/L). Ultrasonography of his testes demonstrated an enlarged left testicle measuring 7 × 5.5 × 4.3 cm with an estimated volume of 87 ml. A large, heterogeneous mass involved the entire testicle with increased vascularity (Fig. 1). The patient’s right testicle measured 3.7 × 2.5 × 1.8 cm with an estimated volume of 8.8 ml. He underwent left orchiectomy. The macroscopic specimen consisted of well-circumscribed nodular lesions of varying sizes, with the largest measuring 45 × 15 mm and containing solid and gelatinous components (Fig. 2). Sections of the specimen showed tumor composed of sheets of small, blue, round cells divided into nodules by fibrous septae (Fig. 3). Immunostaining showed the tumor to be cytokeratin 20 (CK20)-positive with a typical paranuclear dotlike staining pattern (Fig. 4). The stain showed a positive result for CD56, a neuroendocrine marker (Fig. 5), as were CD117 and CK (paranuclear dots). The result was negative for CK7, placental alkaline phosphatase (PLAP), CD30, CD20, AFP, S100, SOX10, prostate-specific antigen (PSA), chromogranin, and thyroid transcription factor 1 (TTF-1). The Ki-67 level was >50 %. This immunostaining pattern raised the possibility of metastatic MCC.

The patient presented ﻿again to our hospital 3 months later this time with a right testicular mass. Tumor markers, including LDH, AFP, and BHCG, were within normal limits. Ultrasonographic imaging demonstrated a new lesion in the right testis measuring 3 × 2.6 × 2.3 cm. He underwent a right orchiectomy, and sections of the specimen showed diffuse infiltration of small, blue, round cells. Immunostains were positive for CD117, CD56, synaptophysin, CK20 (dotlike), and cytokeratin AE1/AE3 (dotlike). The tumor cells were negative for inhibin, PLAP, PSA, S100, CD30, CD45, CD3, CD20, TTF-1, and napsin A. The Ki-67 level was 80 %. This pattern was consistent with a poorly differentiated neuroendocrine tumor in keeping with metastatic MCC. Detailed histology reports can be found in Additional files 2 and 3. A primary site was not identified, and a staging computed tomographic scan did not show evidence of other metastases. The patient received six cycles of adjuvant carboplatin and etoposide chemotherapy. He remained disease-free 18 months following completion of chemotherapy.

## Discussion

It has been suggested that the testes are a sanctuary site for Merkel cell tumors. It is thought that the occurrence of MCC metastases in the testes soon after completion of adjuvant chemotherapy could be due to the presence of a blood-testis barrier that prevents the eradication of tumor cells with chemotherapy agents, thus making the testes a sanctuary site [12]. There are also reports of isolated testicular recurrences in hematological malignancies, suggesting that the testes are a sanctuary site [14, 15]. However, in a retrospective analysis of patients with metastatic germ cell tumors treated with primary chemotherapy, 43 % had no viable tumor in the testes following delayed orchiectomy, showing that adequate concentrations of chemotherapeutic agents can be achieved despite the presence of a blood-testis barrier [16]. It is interesting to note that our patient developed testicular MCC in both testes, with an unknown primary site. It is possible that the primary tumor was eradicated through immunological surveillance, whereas the blood-testis barrier allowed tumor cells in the testes to escape this and to proliferate. Another possibility is that the primary and metastatic disease were heterogeneous and had different properties.

Immunohistochemical analysis helps to differentiate testicular MCC from other malignancies such as metastatic small cell carcinoma of the lung, small cell phenotype melanoma, and lymphoma. Paranuclear dot positivity for CK20 and CAM5.2 is a typical feature of MCC [17]. MCC is also positive for CK AE1/AE3 and neuroendocrine markers, synaptophysin, and chromogranin. Positive CK20 and negative TTF-1 and CK7 distinguish the tumor cells from small cell lung carcinoma [18]. Positive staining for S100, HMB-45, and Melan-A is characteristic of melanoma. Negative leukocyte common antigen (CD45) differentiates MCC from lymphoma.

Treatment of early stage MCC is with surgical resection with 1- to 2-cm margins and sentinel lymph node mapping and biopsy if there is no clinical evidence of regional lymph node involvement. Radical lymphadenectomy is recommended if the regional lymph nodes are involved [19]. Alternatively, definitive lymph node irradiation can be performed because this has been shown to have efficacy similar to completion lymphadenectomy, with no difference in overall survival [20]. In a retrospective analysis of 6908 patients with MCC, adjuvant radiation to the resection site was associated with improved overall survival for stage I-II MCC, whereas adjuvant chemotherapy did not improve overall survival for stage III MCC [21]. In another population study of 4815 patients with head and neck MCC, adjuvant chemoradiation and radiation therapy conferred better overall survival than surgery alone. A survival benefit was also demonstrated in the adjuvant chemoradiation group compared with the adjuvant radiotherapy group in patients with tumor sizes of at least 3 cm, positive margins, and male sex [22].

Chemotherapy regimens for metastatic MCC are often similar to the ones used for high-grade neuroendocrine cancers and small cell cancers, with a number of agents showing activity, including combinations of cisplatin, carboplatin, etoposide, cyclophosphamide, doxorubicin, vincristine, bleomycin, and 5-fluorouracil. MCC is regarded as chemotherapy-sensitive, but the duration of response is short. In one series, the response rates to chemotherapy were 57 % for metastatic MCC and 69 % for locally advanced MCC, with median overall survival of 9 months and 24 months, respectively [23]. The main predictor of survival in MCC is tumor stage at the time of diagnosis. Male sex and tumor size greater than 2 cm are unfavorable factors [1]. It has been suggested that low tumor depth, absence of lymphovascular invasion, a nodular growth pattern, and intratumoral lymphocyte infiltration are associated with a lower risk of death [24, 25]. It has also been noted that patients with MCPyV DNA-positive tumors have a favorable prognosis compared with those with MCPyV DNA-negative tumors [26].

## Conclusions

We present a rare case of a patient with testicular MCC occurring twice with an occult primary tumor. Although a treatment guideline for MCC has been proposed [19], there is no guidance on how to manage testicular MCC. Current treatment is individualized and depends on the extent of disease at the time of diagnosis. Orchiectomy is the initial management modality if the disease is localized to the testes, but the role of adjuvant therapy is yet to be determined.

## Additional files

Additional file 1: Table S1. Clinical details of all known cases of testicular Merkel cell carcinoma. (XLSX 11 kb)

Additional file 2: Supplemental information on histology 1. (DOC 29 kb)

Additional file 3: Supplemental information on histology 2. (DOC 25 kb)

## Abbreviations

AFP: Alpha-fetoprotein
BHCG: Beta-human chorionic gonadotropin
CK: Cytokeratin
LDH: Lactate dehydrogenase
MCC: Merkel cell carcinoma
MCPyV: Merkel cell polyoma virus
PLAP: Placental alkaline phosphatase
PSA: Prostate-specific antigen
TTF-1: Thyroid transcription factor 1

## References

[1] Albores-Saavedra J, Batich K, Chabo-Montero F, Sagy N, Schwartz AM, Henson DE. Merkel cell carcinoma demographics, morphology, and survival based on 3870 cases: a population based study. J Cutan Pathol. 2010;37:20–7. 10.1111/j.1600-0560.2009.01370.x 19638070

[2] Clarke CA, Robbins HA, Tatalovich Z, Lynch CF, Pawlish KS, Finch JL, *et al*. Risk of Merkel cell carcinoma after solid organ transplantation. J Natl Cancer Inst. 2015;107:dju382. 10.1093/jnci/dju382 PMC4311175 25575645

[3] Tadmor T, Aviv A, Polliack A. Merkel cell carcinoma, chronic lymphocytic leukemia and other lymphoproliferative disorders: an old bond with possible new viral ties. Ann Oncol. 2011;22:250–6. 10.1093/annonc/mdq308 20587511

[4] Engels EA, Frisch M, Goedert JJ, Biggar RJ, Millar RW. Merkel cell carcinoma and HIV infection. Lancet. 2002;359:497–8. 10.1016/S0140-6736(02)07668-7 11853800

[5] Tilling T, Moll I. Which are the cells of origin in Merkel cell carcinoma? J Skin Cancer. 2012;2012:680410. doi:10.1155/2012/680410. PMC3530849 23304516

[6] Zur Hausen A, Rennspiess D, Winnepenninckx V, Speel EJ, Kurz AK. Early B-cell differentiation in Merkel cell carcinomas: clues to cellular ancestry. Cancer Res. 2013;73:4982–7. 10.1158/0008-5472.CAN-13-0616 23576560

[7] Feng H, Shuda M, Chang Y, Moore PS. Clonal integration of a polyomavirus in human Merkel cell carcinoma. Science. 2008;319:1096–100. 10.1126/science.1152586 PMC2740911 18202256

[8] Gleason JM, Köhler TS, Monga M. Merkel cell carcinoma metastatic to testis. Urology. 2006;67:423.e13–e14. 10.1016/j.urology.2005.08.043 16461106

[9] Ro JY, Ayala AG, Tetu B, Ordonez NG, el-Naggar A, Grignon DJ, *et al*. Merkel cell carcinoma metastatic to the testis. Am J Clin Pathol. 1990;94:384–9. 10.1093/ajcp/94.4.384 1699405

[10] Rufini V, Perotti G, Brunetti M, Crescenzi A, Fadda G, Troncone L. Unsuspected testicular metastases from Merkel cell carcinoma: a case report with therapeutic implications. Am J Clin Oncol. 2004;27:636–7. 10.1097/01.coc.0000146413.25203.0d 15577445

[11] Schwindl B, Meissner A, Giedl J, Klotz T. Merkel cell carcinoma – a rarity in the urogenital tract. Onkologie. 2006;29:326–8. 10.1159/000093661 16874017

[12] Tummala MK, Hausner PF, McGuire WP, Gipson T, Berkman A. Testis: a sanctuary site in Merkel cell carcinoma. J Clin Oncol. 2006;24:1008–9. 10.1200/JCO.2005.01.7103 16484705

[13] Whitman EJ, Brassell SA, Rosner IL, Moncur JT. Merkel cell carcinoma as a solitary metastasis to the testis. J Clin Oncol. 2007;25:3785–6. 10.1200/JCO.2007.12.3091 17704430

[14] Finklestein JF, Miller DR, Feusner J, Stram DO, Baum E, Shina DC. Treatment of overt isolated testicular relapse in children on therapy for acute lymphoblastic leukemia: a report from the Children’s Cancer Group. Cancer. 1994;73:219–23. 10.1002/1097-0142(19940101)73:1<219::aid-cncr2820730137>3.0.co;2-2 8275428

[15] Locatelli F, Schrappe M, Bernardo ME, Rutella S. How I treat relapsed childhood acute lymphoblastic leukemia. Blood. 2012;120:2807–16. 10.1182/blood-2012-02-265884 22896001

[16] Leibovitch I, Little Jr JS, Foster RS, Rowland RG, Bihrle R, Donohue JP. Delayed orchiectomy after chemotherapy for metastatic nonseminomatous germ cell tumors. J Urol. 1996;155:952–4. 8583615

[17] Aron M, Zhou M. Merkel cell carcinoma of the genitourinary tract. Arch Pathol Lab Med. 2011;135:1067–71. 10.5858/2010-0072-RSR2 21810003

[18] Byrd-Gloster AL, Khoor A, Glass LF, Messina JL, Whitsett JA, Livingston SK, *et al*. Differential expression of thyroid transcription factor 1 in small cell lung carcinoma and Merkel cell tumor. Hum Pathol. 2000;31:58–62. 10.1016/s0046-8177(00)80199-9 10665914

[19] Lebbe C, Becker JC, Grob JJ, Malvehy J, Del Marmol V, Pehamberger H, *et al*. Diagnosis and treatment of Merkel cell carcinoma: European consensus-based interdisciplinary guideline. Eur J Cancer. 2015;51:2396–403. 10.1016/j.ejca.2015.06.131 26257075

[20] Fang LC, Lemos B, Douglas J, Iyer J, Nghiem P. Radiation monotherapy as regional treatment for lymph node positive Merkel cell carcinoma. Cancer. 2010;116:1783–90. 10.1002/cncr.24919 PMC9439996 20162707

[21] Bhatia S, Iyer JG, Storer B, Moshiri A, Parvathaneni U, Byrd DR, *et al*. Adjuvant radiation therapy and chemotherapy in Merkel cell carcinoma: survival analysis of 6,908 cases from the National Cancer Data Base [abstract]. J Clin Oncol. 2014;32(15 Suppl):9014.

[22] Chen MM, Roman SA, Sosa JA, Judson BL. The role of adjuvant therapy in the management of head and neck Merkel cell carcinoma: an analysis of 4815 patients. JAMA Otolaryngol Head Neck Surg. 2015;141:137–41. 10.1001/jamaoto.2014.3052 25474617

[23] Voog E, Biron P, Martin JP, Blay JI. Chemotherapy for patients with locally advanced or metastatic Merkel cell carcinoma. Cancer. 1999;85:2589–95. 10.1002/(sici)1097-0142(19990615)85:12<2589::aid-cncr15>3.0.co;2-f 10375107

[24] Andea AA, Coit DG, Amin B, Busam KJ. Merkel cell carcinoma: histologic features and prognosis. Cancer. 2008;113:2549–58. 10.1002/cncr.23874 18798233

[25] Paulson KG, Iyer JG, Tegeder AR, Thibodeau R, Schelter J, Koba S, *et al*. Transcriptome-wide studies of Merkel cell carcinoma and validation of intratumoral CD8^+^ lymphocyte invasion as an independent predictor of survival. J Clin Oncol. 2011;29:1539–46. 10.1200/JCO.2010.30.6308 PMC3082974 21422430

[26] Sihto H, Kukko H, Koljonen V, Sankila R, Bohling T, Joensuu H. Clinical factors associated with Merkel cell polyomavirus infection in Merkel cell carcinoma. J Natl Cancer Inst. 2009;101:938–45. 10.1093/jnci/djp139 19535775

