# Supplementary material for: Recurrent Merkel cell carcinoma of the testis with unknown primary site: a case report
Source: J Med Case Rep. 2016 Nov 5;10:314. doi: 10.1186/s13256-016-1102-5 (PMC5097413; doi:10.1186/s13256-016-1102-5)
Supplement: Additional file 2: — Supplemental information on histology 1. (DOC 29 kb) [file 13256_2016_1102_MOESM2_ESM.doc]

| | Clinical Details: L testicular mass, normal AFP, normal BHCG, LDH>200, US - L testicular mass, L radical orchidectomy + excision of lipoma of the cord.   Specimen A: TESTIS, ORCHIDECTOMY                                           The specimen container is labelled "left testicle".   Macroscopic:     The specimen consists of a testicle with attached tunica vaginalis.  The specimen measures 85 x 75 x 45mm and weighs 140.5gm.  A photograph of the specimen is attached.  On bisecting, the interior is composed of well circumscribed nodular lesions of varying sizes. The largest nodule measures 45 x 15mm and contains both solid and gelatinous-like components.  A photograph of the bisected specimen is attached.    Block Description:   A1 = cross section through the cord   A2 = section through the rete testes   A3 = section from the largest nodule including the gelatinous region   A4 = section where the tunica is adherent to the testicle A5-A6 = further representative sections through the lesion    Microscopic:    Sections of the tumour show a tumour composed of sheets of small blue round cells divided into nodules by fibrous septae. The cells have scant cytoplasm, round to oval nuclei with dense granular chromatin and inconspicuous nucleoli. Scattered apoptotic cells are seen and numerous mitoses are also seen. There are central areas of haemorrhage and necrosis. The tumour extends into but not through the tunica. There is expansion into the rete. No convincing intratubular germ cell neoplasia is seen. The spermatic cord and cord margin are not involved. A special stain for PAS is negative.  Immunostains show the tumour to be: Positive - CD117 (focal), CK (paranuclear dots), CK20 (paranuclear dots), CD56 Negative - CK7, PLAP, CD30, CD20, AFP, S100, SOX10, chromogranin, TTF-1 Ki-67 proliferation is elevated >50%  The appearances are those of a poorly differentiated neuroendocrine carcinoma with small cell pattern. The negative TTF-1 counters a diagnosis of typical small cell carcinoma, whilst the paranuclear dot positive staining pattern with CK20 raises the possibility of a metastatic Merkel cell carcinoma.   CONCLUSION:  LEFT TESTICLE, RADICAL ORCHIDECTOMY - POORLY DIFFERENTIATED NEUROENDOCRINE CARCINOMA, SEE TEXT | | --- | |
| --- | --- |
