# Supplementary material for: Recurrent Merkel cell carcinoma of the testis with unknown primary site: a case report
Source: J Med Case Rep. 2016 Nov 5;10:314. doi: 10.1186/s13256-016-1102-5 (PMC5097413; doi:10.1186/s13256-016-1102-5)
Supplement: Additional file 3: — Supplemental information on histology 2. (DOC 25 kb) [file 13256_2016_1102_MOESM3_ESM.doc]

Clinical Details:
Right inguinal orchiectomy for ?tumour relaying testicle on US. Left inguinal orchiectomy in April 2014 -> poorly differentiated neuroendocrine.
R testis tumour was normal at that stage.

Specimen A: TESTIS, ORCHIDECTOMY
The specimen container is labelled "right testicle".

Macroscopic:    
The specimen consists of a testis measuring 65x50x35mm. There is attached spermatic cord measuring 60x15x7mm. The specimen weighs 110gm. The exterior is marked with blue ink and the spermatic cord resection margin is marked with yellow ink. On bivalving, the testis is filled with a cream and haemorrhagic mass. Mass occupies the entire testis and measures up to 55mm in maximum dimension.  The cut surface is photographed.

Block Description
  A1 = spermatic cord resection margins.
  A2 = section from mid point of spermatic cord.
A3-A8 = representative sections of the tumour and adjacent tissue.

Microscopic:  
Sections show testis which is almost completely replaced by a diffuse infiltration of small round blue cells. The tumour cells are separated into irregular nodules by fibrous septae. Areas of necrosis and haemorrhage are noted. The tumour cells show scant cytoplasm, round-to-oval nuclei and granular chromatin with inconspicuous nucleoli. Numerous mitotic figures are noted (>7 per 10 high power field). The tumour does not appear to penetrate through the tunica albuginea. There is no evidence of intra-tubular germ cell neoplasia. The spermatic cord is not involved at the resection margins.

The tumour cells show positivity with CD117, CD56, synaptophysin, CK20 (dot like) and cytokeratin AE1/AE3 (dot like).

The tumour cells are negative inhibin, PLAP, prostate specific antigen, S100, CD30, CD45, CD3, CD20, TTF1 and Napsin A.

Ki67 shows a high proliferative index of approximately 80%.

The appearances are poorly differentiated neuroendocrine carcinoma. The possibility of metastasis from a Merkel cell carcinoma should be considered. The differential diagnosis include metastasis from other primary sites e.g. lung. However, this is considered to be less likely. Clinico-pathological correlation is required.

CONCLUSION:
RIGHT TESTICLE - POORLY DIFFERENTIATED NEUROENDOCRINE CARCINOMA. METASTASIS FROM A MERKEL CELL CARCINOMA SHOULD BE EXCLUDED. CLINICO-PATHOLOGICAL CORRELATION IS REQUIRED.
